# Circular RNA expression profiles during the differentiation of mouse neural stem cells

**DOI:** 10.1186/s12918-018-0651-1

**Published:** 2018-12-21

**Authors:** Qichang Yang, Jing Wu, Jian Zhao, Tianyi Xu, Zhongming Zhao, Xiaofeng Song, Ping Han

**Affiliations:** 1 Department of Biomedical Engineering, Nanjing University of Aeronautics and Astronautics, Nanjing, 211106 Jiangsu China; 2 Center for Precision Health, School of Biomedical Informatics, The University of Texas Health Science Center at Houston, Houston, TX 77030 USA; 3 Department of Biomedical Informatics, Vanderbilt University Medical Center, Nashville, TN 37203 USA; 4 The First Affiliated Hospital with Nanjing Medical University, Nanjing, 210019 Jiangsu China

**Keywords:** Circular RNA;neural stem cell, Differentiation, Expression profile, Gene regulation

## Abstract

**Background:**

Circular RNAs (circRNAs) have recently been found to be expressed in human brain tissue, and many lines ofevidence indicate that circRNAs play regulatory roles in neurodevelopment. Proliferation and differentiation of neural stem cells (NSCs) are critical parts during development of central nervous system (CNS).To date, there have been no reports ofcircRNA expression profiles during the differentiation of mouse NSCs. We hypothesizethat circRNAs mayregulate gene expression in the proliferation anddifferentiation of NSCs.

**Results:**

In this study, we obtained NSCs from the wild-type C57BL/6 J mouse fetal cerebral cortex. We extracted total RNA from NSCs in different differentiation stagesand then performed RNA-seq. By analyzing the RNA-Seq data, we found 37circRNAs and 4182 mRNAs differentially expressedduringthe NSC differentiation. Gene Ontology (GO) enrichment analysis of thecognate linear genes of these circRNAsrevealed that some enriched GO terms were related to neural activity. Furthermore, we performed a co-expression network analysis of these differentially expressed circRNAs and mRNAs. The result suggested a stronger GO enrichmentin neural features for both the cognate linear genes of circRNAs and differentially expressed mRNAs.

**Conclusion:**

We performed the first circRNA investigation during the differentiation of mouse NSCs. Wefound that12 circRNAs might have regulatory roles duringthe NSC differentiation, indicating that circRNAs might be modulated during NSC differentiation.Our network analysis suggested the possible complex circRNA-mRNA mechanisms during differentiation, and future experimental workis need to validate these possible mechanisms.

**Electronic supplementary material:**

The online version of this article (10.1186/s12918-018-0651-1) contains supplementary material, which is available to authorized users.

## Background

Canonical RNA had been known asa linear molecule that terminates in 5′ cap and 3′ poly(A) tail. However, in 1976 a new class of endogenous circular RNA (circRNA) was discovered in a viroid [1]. Since the first discovery, many circRNAshave been identified and they present in all eukaryotes ranging from yeast, fruit flies (*Drosophila*), to humans [2, 3]. As a class of non-coding RNAs, circRNAs are characterized by covalently closed continuous loop. Due to the 5′ and 3′ end deficiency, circRNAs cannot be degraded by RNase R and present permanent stability in cells [4]. For many years after 1976, there had been only a few circRNAs whose expression was experimentally validated in cells [5]. However, the recent application of innovative, high-throughput sequencing technologies allowed investigators to discover tens of thousands of circRNAs from poly (A)-minus or RNase R treated RNA-Seq data [6, 7]. In the past a few years, circRNAs increasingly attractedresearchers’ attention and re-emerged as a burgeoning class of non-coding RNAs [8].

With the accelerating generation of RNA-Seq data, more and more cellular and functional circular RNAshave been recently reported in different cell types and tissues [9]. In human neural tissue, circRNAs were foundto play a functional role in maintenance, plasticity and abnormality of neural circuitry [10, 11]. For instance, Yang and his colleagues reported that circZNF292 silencing could suppress the proliferative and angiogenic potential of glioma cells, suggesting that circRNAsmight regulate many biological processes [12]. Recently, many lines of evidence have unveiled that circRNAs are enriched in brain tissuewhen compared with their expressions in other tissues [13]. Investigators also found that while most circRNAs are inlow expression level, they are conserved and expressed in a time-, cell type- andgene-specific manner [14]. Cognate genesrefer to those linear genes have the same sequences with circRNAs. Some circRNAs are present at higher copynumbers when compared to their cognate genes (> 10-fold) [15].So far, the function of most circRNAshas still been elusive. One common function is that some circRNAs can act their roles as microRNA (miRNA) ‘sponges’ – sponge RNAs containing complementary binding sites to a miRNA of interest so that theyprevent miRNAs from the connections with the target mRNA [16, 17]. Some circRNAs can also regulate miRNAs through RNA-binding proteins (RBPs) and affect cellular function, indicating another important function in the interaction with miRNAs [18]. Although functional studies have been reported in literature, investigation ofcircRNA’sfunction in neural tissue has not been systematically done yet.

Central nervous system (CNS) is a significant part of neurodevelopment, which contains brain and spinal cord. The CNS development starts in early embryo and plays an extremely important role in human development from fetal to adult stages. In recent years, studies showed that circRNAs have close connection with neuronal development. CircRNAs are not expressed equally in the CNS, rather, they are differentially distributed in different brain regions and different developmental stages [19]. For example, Vanet al. found a striking regulation of circRNAs during neuronal development [20, 21]. In the meanwhile, several circRNAswere found to be expressed at high level in the cerebellum when compared withthe brainstem [17]. A large amount ofcircRNAswere detected in synapses and their regulations were also confirmed [22, 23]. Many circRNAswereexperimentally verified in different cell typesin mice [24]. Zhao et al. reported that circRNAsmight have physiological functions in neuron, andsome of these circRNAswere related tobrain disease [25]. Many brain diseases are attributed to the abnormal development of CNS [26]. For example, dysplasia in CNS leads to many cerebral diseases, such as CNS tumor in childhood, and Alzheimer’s and Parkinson’s in elder people [27, 28]. Some studies indicated that over-expressed circRNAsmight initiate the occurrence of neurological diseases [29]. However, the expression pattern and function of circRNAs in the brain diseases or CNS development are still largely unclear.

Recently, several circRNAs were found to involve in neural function.For example, twocircRNAs, circRNAsex-determining region Y(*SRY*) and cerebellar degeneration-related protein 1 antisense(*CDR1as*) have several binding sites with *miR-138* and *miR-7*, respectively [30, 31]*.* By binding miRNA, these circRNAscould regulate the expression of miRNA and furthermore suppress their function, which is known as ‘sponging RNA’ [32]. And *circ-HIPK3* was shown to bind with miR-24 and furthermore modulate human cell growth [33]. While these circRNAs act as competitive RNAs for miRNAs, other studies suggested their connection with RBPs as well. CircRNAs from the muscle blind (*mbl*) and *FOXO3* genes were reported to bind,sequester andtransport RBPs [34]. The researchers thought these circRNAs might regulate the interaction of RBPs with their RNAtargets [35]. Some other studies revealed that alternativesplicing of circRNAs might also lead to the newbinding sequences for some RBPs and,thus, influence functions [36]. So far, it is not clear whether miRNA sponging andRBP binding are shared functions of circRNAs.

In this work, we examined circRNA expression pattern during the NSC differentiation by analyzing the RNA-Seq data. Previous experimental results reported that at the time course of differentiation day 2, the NSC activation reached their peaks and the NSC differentiation was also strongly activated [37, 38]. In this study, we identified differentially expressed circRNAs and their cognate linear mRNAs in different differentiation stages.There wasa total of 37 circRNAs differentially expressed in this process. It is likely some of thesecircRNAsinvolved in differentiation and resulted in the corresponding expression profiles. Further analysis of the expression profiles during NSCdifferentiation could help us uncover the possible regulatory circRNAs. We further found out the differentially expressed mRNAs and then constructed a co-expression network between these potentially regulatory circRNAs and differentially expressed mRNAs by the same binding miRNA. The Gene Ontology (GO) enrichment analysis on them suggested stronger enrichment in neural features and pointed outa possible regulation of circRNAsduring the differentiation.Furthermore, the opposite expression patterns between circRNAs and mRNAs suggested complex circRNA-mRNA mechanisms in the NSC differentiation.

## Results

### CircRNAsare abundant and highly expressed in NSC differentiation

**Fig. 1:**
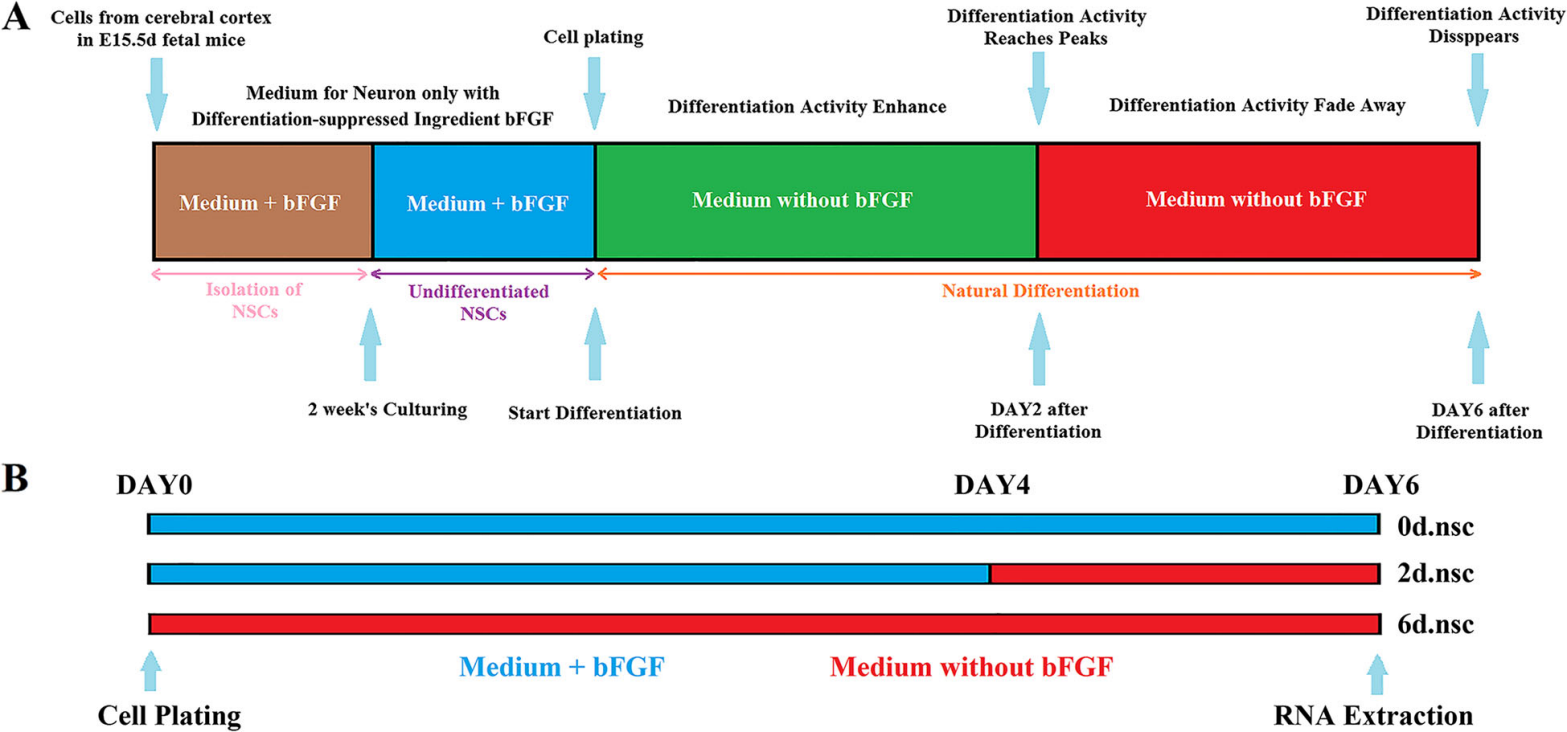
Flow chart of the experiment. **a** The left flow chart explains the process for isolation, culture and differentiation of NSCs. **b** The right flow chart explains the different differentiation time in the triple groups of NSCs

Total RNA was collected from mouse NSCs cultured in differentiation-suppressed medium or induced to differentiation with two replicates (Fig. 1). One group of NSCs wasculturedand kept undifferentiated with the differentiation-suppress ingredient bFGFin 6 days as 0d.nsc group. In the 2d.nsc group, NSCs were first kept undifferentiated in 4 days and then induced to differentiation in 2 days without adding bFGF.And in the 6d.nsc group, NSCs were induced at the beginning of culturing and were kept in differentiation state in 6 days. Paired-end ribominus RNA sequencing (RNA-Seq) was performed, and the UROBORUS computational pipeline was applied to detect potential circRNAs in differentiation [39]. Firstly, RNA-Seq data were mapped to reference genomeusing toolTopHat (version 2.1.1). The transcriptome was then reconstituted with Cufflinks(version 2.2.1) (Additional file 1: Table S1). Welisted the top 200 circular RNA genes expressed in eachgroup, and presented their pair-wise expressioncorrelationmatrix resultsofthese 200circular RNAgenes (shown in Additional file 1: Figure S1). The correlation values ofthese three groupswere allabove 0.94, indicating the strong consistencyand reproducibility.Moreover, the moderate correlations (0.68–0.84)in2d.nsc versus 0d.nsc and 6d.nsc versus 2d.nscindicated their close relationship but withdifferent expressionpatterns. In the comparison of the 6d.nsc and 0d.nsc groups, the correlations were among 0.51–0.57. The weaker correlations suggested strongerdifferentiation-related circular RNA expression variation between groups6d.nsc and 0d.nsc than that between groups 2d.nsc and 0d.nsc, or between groups6d.nsc and 2d.nsc. Differential linear RNAexpression analysis was further performed to check the quality of the RNA-Seq data in NSCdifferentiation experiments. The comparison between groups2d.nsc and0d.nscidentified2917linear RNAs weresignificantly upregulated or downregulated in the NSC differentiation process, and2360differentially expressedlinear RNAsbetween groups6d.nsc and 2d.nsc (Additional file 1: Table S2 and Figure S2). We performed GO enrichment analysis onthese linear RNAs. The resultsindicated the consistent and significant enrichment of these genes in function that is related to neurodevelopment, neural activity, and differentiation (Additional file 1: Figure S3). Moreover, the expression of well-known specific markers of neurodevelopment, neural activity or differentiation was also found in the differentially expressedgene list, furtherhighlighting the regulatory function of these differentially expressed mRNA during the differentiation.

**Fig. 2:**
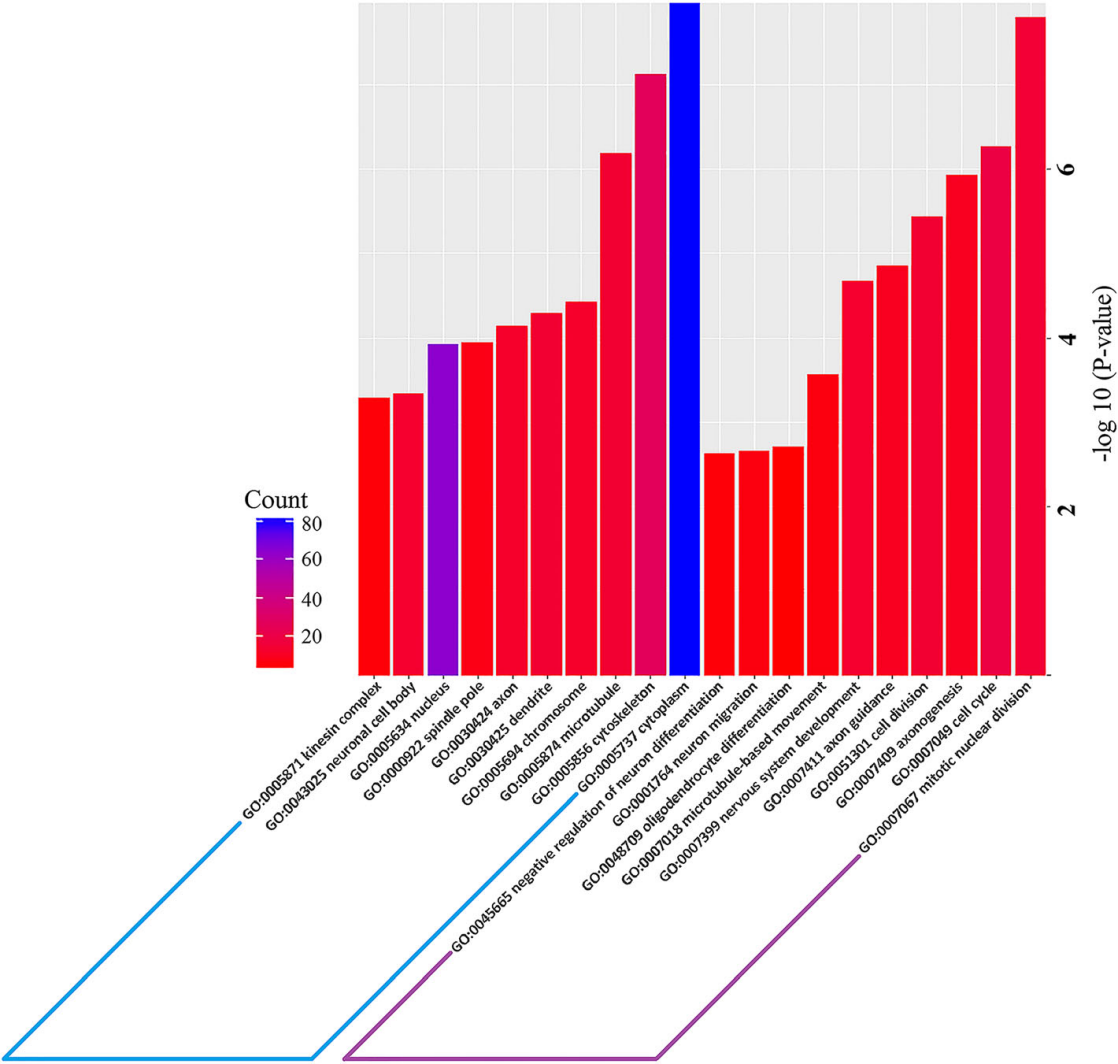
GO enrichment analysis of the differentially expressed mRNA in the Pattern 4. The GO analysis (*P*-value < 0.05 and supporting reads number > =2) contains two domains: Biological Processand Cellular Component

To further find the specific mRNAs with regulatory function during the differentiation, we found1173 mRNAs that weredifferentially expressed in both differential expression analysis between2d.nsc versus 0d.nsc and 6d.nsc versus 2d.nsc.That is, these mRNAs were differentially expressed during the whole NSC differentiation. Then, we separatedthese differentially expressedmRNAs into four expressionpatterns for better illustration in further analysis. Pattern 1 consisted ofthose mRNAs with their expression upregulated;we named this expression profile as0d.nsc < 2d.nsc and 2d.nsc < 6d.nscduringthe differentiation. Pattern 2 contained those mRNAs with the opposite expression against pattern 1.Pattern 3 consisted of mRNAs with expression profileas0d.nsc < 2d.nsc and 2d.nsc > 6d.nsc. And similarly, pattern 4 showed the opposite trend ofPattern 3. We separately applied GO and Kyoto Encyclopedia of Genes and Genomes (KEGG)pathway enrichment analyses on these differentially expressed mRNAsduring the differentiation (Fig. 2 and Additional file 1: Figure S4). As summarized in Additional file 1: Table S3, these analyses consistently revealed the enriched GO terms much related to neural activitywith one exception (the results between Pattern 4 with other Patterns). Patterns 1, 2 and 3 had several irrelevant GO enrichment terms in their top lists while the top GO enrichment terms in Pattern 4 were most connected to the neural activity or development of CNS. This observation suggested thatmore mRNAs with lower expression in 2d.nsc had regulatory function in differentiation. When we traced back to the number of expressed mRNAs in the different groups, we found that the number of mRNAs is the lowest when the threshold was set to FPKM> 10 or FPKM > 100 (Additional file 1: Table S1). In the previous GO enrichment analysis, we also discovered that theenriched GO terms from down-regulated mRNAs in 2d.nsc versus 0d.nsc and up-regulated mRNA in 6d.nsc versus 2d.nscweremore associated with neural activity than those up-regulated mRNAs in 2d.nsc versus 0d.nsc and down-regulated mRNA in 6d.nsc versus 2d.nsc, which coincided with the variation in Pattern 4 (Additional file 1: Figure S3). This phenomenon suggested that there were fewer mRNAs expressed in the medium differentiation stage, and those mRNAs in Pattern 4(i.e., expression profile in 0d.nsc > 2d.nscand 2d.nsc < 6d.nsc) might more involve in neuronal related functions during the differentiation of NSCs.

In the next step, RNA-Seqshort reads were aligned to the reference genome (version GRCm38/mm10) and the UROBORUS computational pipeline was applied to detect circRNAs in differentiation. More than 4000 potential circRNAs were found in every group (Additional file 1: Table S4). We listed the top 100 expressed circRNAs and found that all these circRNAswere expressed in tissues from mouse brain based on our search of the database circPedia. This additional information preliminarily confirmed the expression feature of circRNAs detected in the NSC differentiationin our study. We further checked the function of their cognate genesusing UCSC (The University of California Santa Cruz) Genome Browser. We found many of them have regulations in development and neural activity. In the next, we further explored whether these circRNAshave regulatory function during NSC differentiation.

### CircRNAexpression is modulated during NSC differentiation

**Fig. 3:**
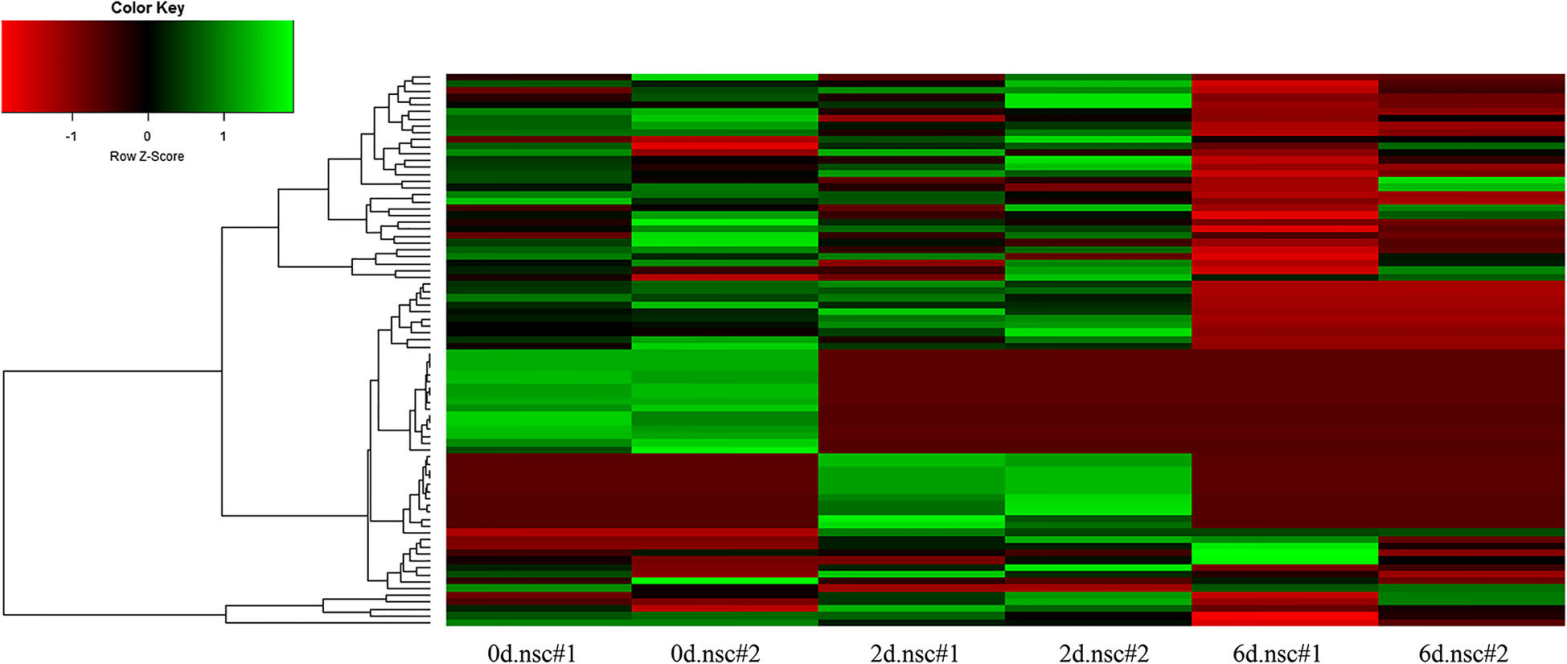
Expression profiling of circRNAs detected during NSC differentiation. Heatmap of expression profiles for circRNAs that were detected during the differentiation (68 circRNAs in 0d.nsc, 64 circRNAs in 2d.nsc and 43 circRNAs in 6d.nsc), red to green color bar indicates the expression level from low to high

**Fig. 4:**
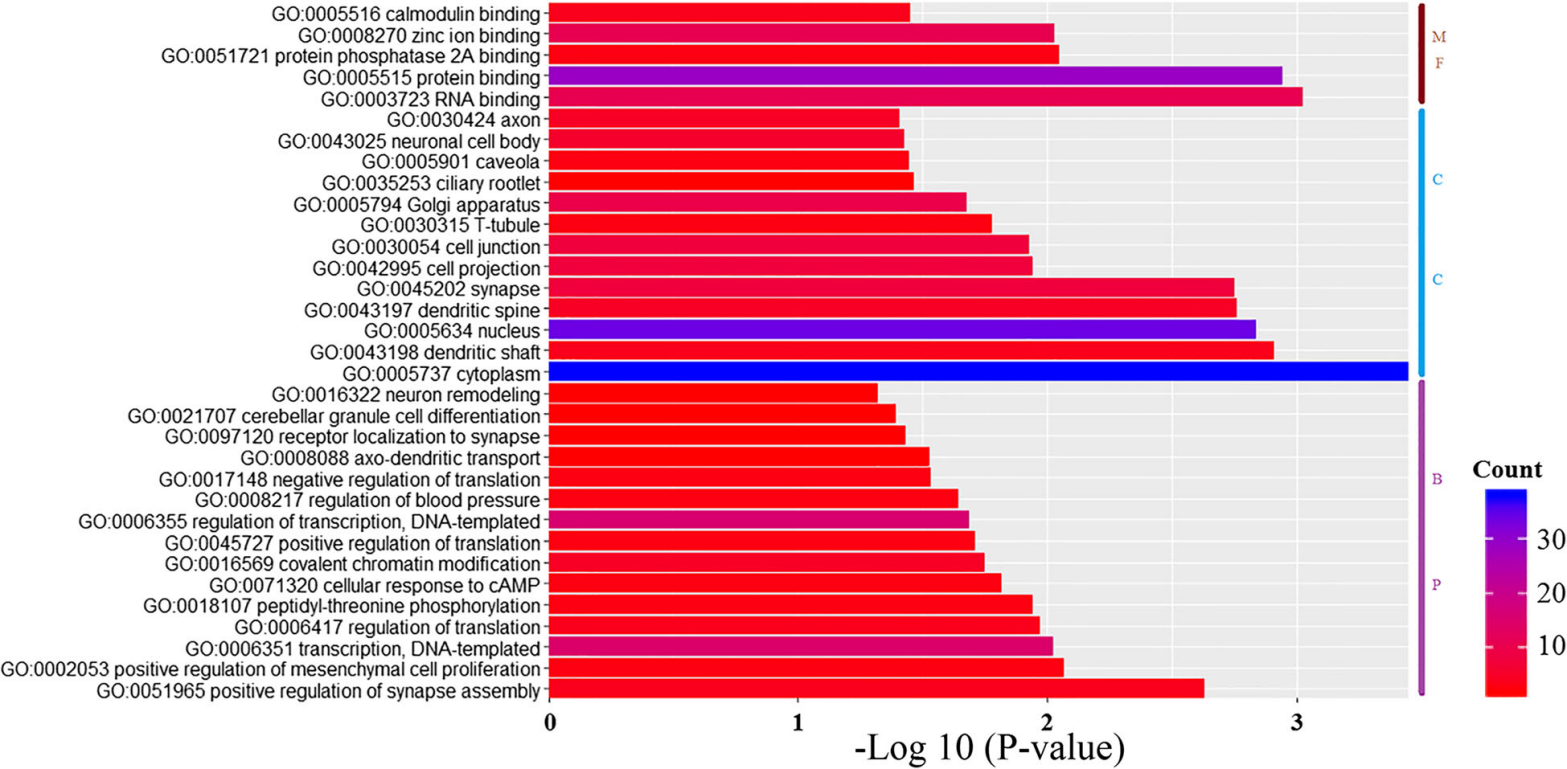
GO enrichment analysis of the cognate genes of the circRNAs detected during the differentiation. The GO analysis (*P*-value < 0.05 and supporting reads number > =2) contains three domains: Biological Process, Cellular Component and Molecular Function. GO enrichment analysis on the cognate genes of the circRNAs detected expressing in all groups

To perform quantitative analysis ofcircRNA expression profiles, we firstly filtered out those circRNAs with expression level < 0.1 RPM. Then, we excluded those circRNAs with only expressed in onereplicate. The process resulted in80 circRNAsfor further studies (Fig. 3).All the replicates had an overall better correlation for both circRNAsandtheir cognate mRNAs.CircRNAs had overall correlation coefficients above 0.93 in replicates. And the expressions between these circRNAs and their cognate genes in replicates were compared and the correlation coefficientswere all above 0.92 (Additional file 1: Figure S5). We tested the potential functions of these circRNAs by gene set enrichment analysis. We first listed the cognate genes of these circRNAs and then applied GO enrichment analysis of them (Fig. 4).

For GO analysis, genes were organized into hierarchical categoriesto discover their regulatory networks based onthree domains: Biological Process, Cellular Component and MolecularFunction. The results showed thatmost of the significant enriched GO terms wererelated to CNS development.We also found that these cognate genes were associated with the following GO Biological Process terms: regulation of synapse assembly, axo-dendritic transport, receptor localization to synapse, cerebellar granule cell differentiation and neuron remodeling; GO Cellular Component terms: dendritic shaft, dendritic spine, synapse, cell projection, cell junction, neuronal cell body and axon; and GO Molecular Function terms: RNA binding and protein binding. These significantly enriched GO terms are closely associated with CNS development, suggesting the potential connections between these circRNAs and CNS development. Taken together, these results suggested that the circRNAs we identified might have close connection with the neural activity.

**Fig. 5:**
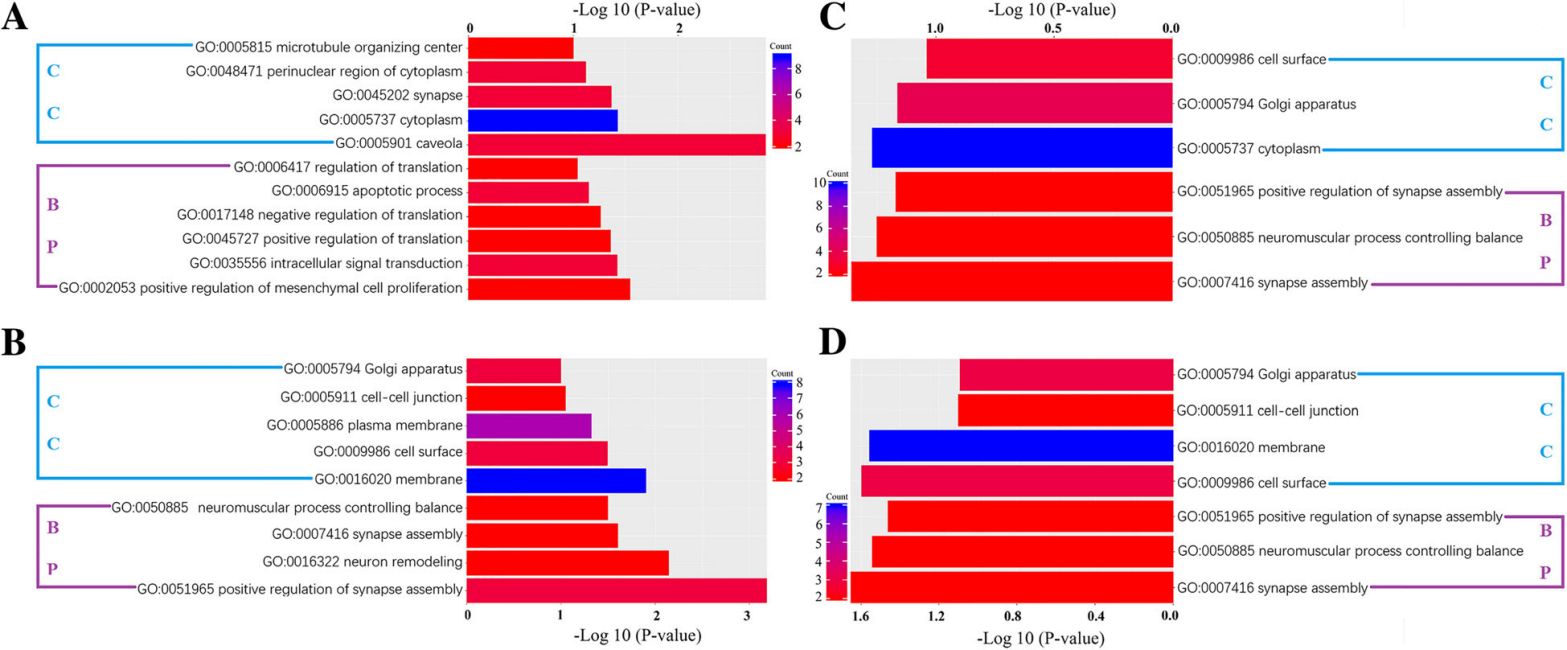
GO enrichment analysis of the cognate genes from the differentially expressedcircRNAs. The GO analysis (*P*-value < 0.05 and supporting reads number > =2) contains two domains: Biological Process andCellular Component. **a** Down-regulated circRNAs in 2d.nsc versus 0d.nsc. **b** Up-regulated circRNAs in 2d.nsc versus 0d.nsc. **c** Down-regulated circRNAs in 6d.nsc versus 2d.nsc. **d** 11 differentially expressedcircRNAs in both 2d.nsc versus 0d.nsc and 6d.nsc versus 2d.nsc

The comparison of circRNA expression profiles between 2d.nsc and 0d.nsc and between 6d.nsc and 2d.nsc revealed a global change in circRNA expression, that is, these molecules were likely altered during NSC differentiation (Tables 1 and 2). We identified 28 and 22 circRNAs that were differentially expressed between 2d.nsc and 0d.nsc and between 6d.nsc and 2d.nsc, respectively. We found that 16 circRNAs were upregulated and 12 circRNAs were downregulated in comparison between 2d.nsc versus 0d.nsc. And in the comparison between 6d.nsc and 2d.nsc, 21 circRNAs were downregulated and only 1 circRNA was upregulated. GO enrichment analysis was applied to the cognate genes of these circRNAs: 21 downregulated circRNAsin6d.nsc versus 2d.nsc, 16 upregulated and 12 downregulated circRNAsin2d.nsc versus 0d.nsc (Fig. 5a, b and c). As the results, both downregulated circRNAs in 6d.nsc versus 2d.nsc and upregulated circRNAsin 2d.nscversus 0d.nschad the similar significant enrichment biological process terms, which were also strongly related to NSC differentiation. The GO analysis on downregulated circRNAsin 2d.nscversus 0d.nscindicated the significant enrichment for gene differentiation and translationwhich were alsoenriched in NSC differentiation. Collectively, the resultsabove suggested that circRNA expression is broadly modulated during NSC differentiation and these altered circRNAs are related to neural function.

**Table 1:** Differentially expressed circRNAs in2d.nscversus 0d.nsc

Chromosome	Start junction (bp)	End junction (bp)	Strand	Cognate gene symbol
12 upregulated circRNAs in 2d.nscversus 0d.nsc				
16	85,013,626	85,043,743	–	*App*
17	90,560,740	90,597,629	–	*Nrxn1*
19	28,665,752	28,666,236	–	*Glis3*
17	25,235,181	25,235,627	–	*Gnptg*
5	81,688,508	81,771,680	+	*Adgrl3*
2	68,358,813	68,410,141	–	*Stk39*
16	58,424,560	58,433,463	+	*Dcbld2*
2	33,260,477	33,324,703	–	*Ralgps1*
2	32,182,457	32,194,996	+	*Prrc2b*
1	25,221,759	25,228,547	–	*Adgrb3*
4	84,291,692	84,293,662	–	*Bnc2*
2	68,390,913	68,410,141	–	*Stk39*
16 downregulated circRNAs in 2d.nscversus 0d.nsc				
12	52,516,076	52,542,636	+	*Arhgap5*
2	166,834,470	166,836,457	+	*Arfgef2*
1	185,386,245	185,397,267	+	*Eprs*
7	46,590,606	46,635,556	–	*Sergef*
16	13,697,332	13,699,075	+	*Bfar*
6	134,541,651	134,542,045	–	*Lrp6*
12	53,139,380	53,142,768	+	*Akap6*
2	118,631,758	118,635,277	+	*Bub1b*
13	91,524,584	91,564,694	+	*Ssbp2*
X	69,544,408	69,545,269	+	*Aff2*
14	47,016,276	47,016,795	+	*Samd4*
16	17,015,897	17,018,475	+	*Mapk1*
X	120,399,401	120,401,897	+	*Pcdh11x*
16	30,543,401	30,547,469	–	*Tmem44*
9	22,024,673	22,036,860	–	*Elavl3*
7	18,942,215	18,950,619	+	*Nova2*

**Table 2:** Differentially expressed circRNAs in6d.nscversus 2d.nsc

Chromosome	Start junction (bp)	End junction (bp)	Strand	Cognate gene symbol
1 upregulated circRNA in 6d.nscversus 2d.nsc				
12	52,516,076	52,542,636	+	*Arhgap5*
21 downregulated circRNAs in 6d.nscversus 2d.nsc				
11	31,055,457	31,061,586	+	*Asb3*
12	51,647,993	51,661,713	–	*Strn3*
7	126,551,974	126,552,338	–	*Eif3c*
5	110,755,396	110,756,766	–	*Ep400*
6	88,355,482	88,358,642	–	*Eefsec*
19	57,751,629	57,797,503	+	*Atrnl1*
9	14,571,622	14,575,439	–	*Amotl1*
17	86,098,511	86,120,746	–	*Srbd1*
9	66,540,061	66,566,903	–	*Usp3*
19	57,629,084	57,657,238	+	*Atrnl1*
16	85,013,626	85,043,743	–	*App*
17	90,560,740	90,597,629	–	*Nrxn1*
19	28,665,752	28,666,236	–	*Glis3*
17	25,235,181	25,235,627	–	*Gnptg*
5	81,688,508	81,771,680	+	*Adgrl3*
2	68,358,813	68,410,141	–	*Stk39*
16	58,424,560	58,433,463	+	*Dcbld2*
2	33,260,477	33,324,703	–	*Ralgps1*
2	32,182,457	32,194,996	+	*Prrc2b*
4	84,291,692	84,293,662	–	*Bnc2*
2	68,390,913	68,410,141	–	*Stk39*

### Possible regulation of circRNAsin NSC differentiation

To find the possible regulatory circRNAsduring NSC differentiation, we examinedthe circRNAdifferential expression changesbetween conditions 2d.nsc and 0d.nsc, as well as,between conditions 6d.nsc and 2d.nsc. The results showed that11 circRNAs were upregulated in group 2d.nsc versus 0d.nsc and downregulated in group 6d.nsc versus 2d.nsc while only 1 circRNA had the opposite expression profile, i.e., expression profile in 0d.nsc > 2d.nsc and 2d.nsc < 6d.nsc (Table 3). Interestingly, our GO enrichment analysis on the cognate genes from these 11 circRNAs were consist with the previous GO results (Fig. 5d). These 11circRNAsmay perform function related with NSC differentiation. In our search of thecircPedia database, we found all these circRNAs expressed in tissues of mouse brain. We noted thatonly three circRNAs,*Circ-APP*,*Circ-Arhgap5*and *Circ-Bnc2*, shared the same expression patterns with their cognate linear genes.The remainingcircRNAsmatched only Pattern 4 (expression profile in 0d.nsc < 2d.nscand 2d.nsc > 6d.nsc) and their cognate linear genes were only withPattern 1 (expression profile in 0d.nsc < 2d.nscand 2d.nsc < 6d.nsc).

**Table 3:** 12 circRNAs differentially expressed during the NSC differentiation

Chromosome	Start junction (bp)	End junction (bp)	Strand	Cognate gene symbol	Matched transcript 0d.nsc
12	52,516,076	52,542,636	+	*Arhgap5*	NM_009706
16	85,013,626	85,043,743	–	*App*	NM_007471
17	90,560,740	90,597,629	–	*Nrxn1*	NM_177284
19	28,665,752	28,666,236	–	*Glis3*	NM_175459
17	25,235,181	25,235,627	–	*Gnptg*	NM_172529
5	81,688,508	81,771,680	+	*Adgrl3*	NM_198702
2	68,358,813	68,410,141	–	*Stk39*	NM_016866
16	58,424,560	58,433,463	+	*Dcbld2*	NM_028523
2	33,260,477	33,324,703	–	*Ralgps1*	NM_001290570
2	32,182,457	32,194,996	+	*Prrc2b*	NM_172661
4	84,291,692	84,293,662	–	*Bnc2*	NM_172870
2	68,390,913	68,410,141	–	*Stk39*	NM_016866

We next investigated these 11 circRNAsfor their cognate linear RNAs’ functionfrom previous reports.It has been reported that gene *App* regulates neuronal stem cell differentiation andinduces glial differentiation [40]. The *APPswe/PS1DE9* (*APP/PS1*) mice have been widely used for the study of Alzheimer’s disease due to its lack of *App* protein that contributes to Alzheimer’s disease [41]. *Arhgap5* has no direct function on brain or other neural activity, but it is critical for embryonic mammary bud development [42]. Previous studies have shown that there is significant expression ofPrrc2b protein in developing rat brains [43]. Pak et al. found that *Nrxn1*expression is high during human neocortical development and aging process. Heterozygous mutations in *Nrxn1*can lead to synaptic transmission defects [44]. Kim et al. recently identified that *Glis3*could regulate neurogenin-3 expression in pancreatic β-cells [45]. Acosta et al. applied experiment and confirmed that*Adgrl3* variants are associated with a refined phenotype of attention-deficit/hyperactivity disorder(ADHD) in the MTA study [46]. An ultraconserved brain-specific enhancer in *ADGRL3*were reported withADHD susceptibility [47].*STK39*has been reported as associated with Parkinson’s Disease and has regulation in cell invasion and proliferation [48]. Besides, several groups reported that *Dcbld2* is one identification of novel neuroendocrine-specific tumor genes and regulates gastric cancer cell proliferation and invasion [49].Collectively, these previous studies supported our analysis results that circRNAs can performs similar function because the circular and linear RNA were homologous. There has been no direct evidence of*Gnptg*,*Ralgps1* and *Bnc2*in CNS development or involvement in other neural activity, but our resultsindicated that the circular isoforms from these three genes have potential function during NSCs differentiation. This novel finding warrants further investigation in future and we will keep studying on them.

### Construction of the circRNA-mRNA co-expression network

**Fig. 6:**
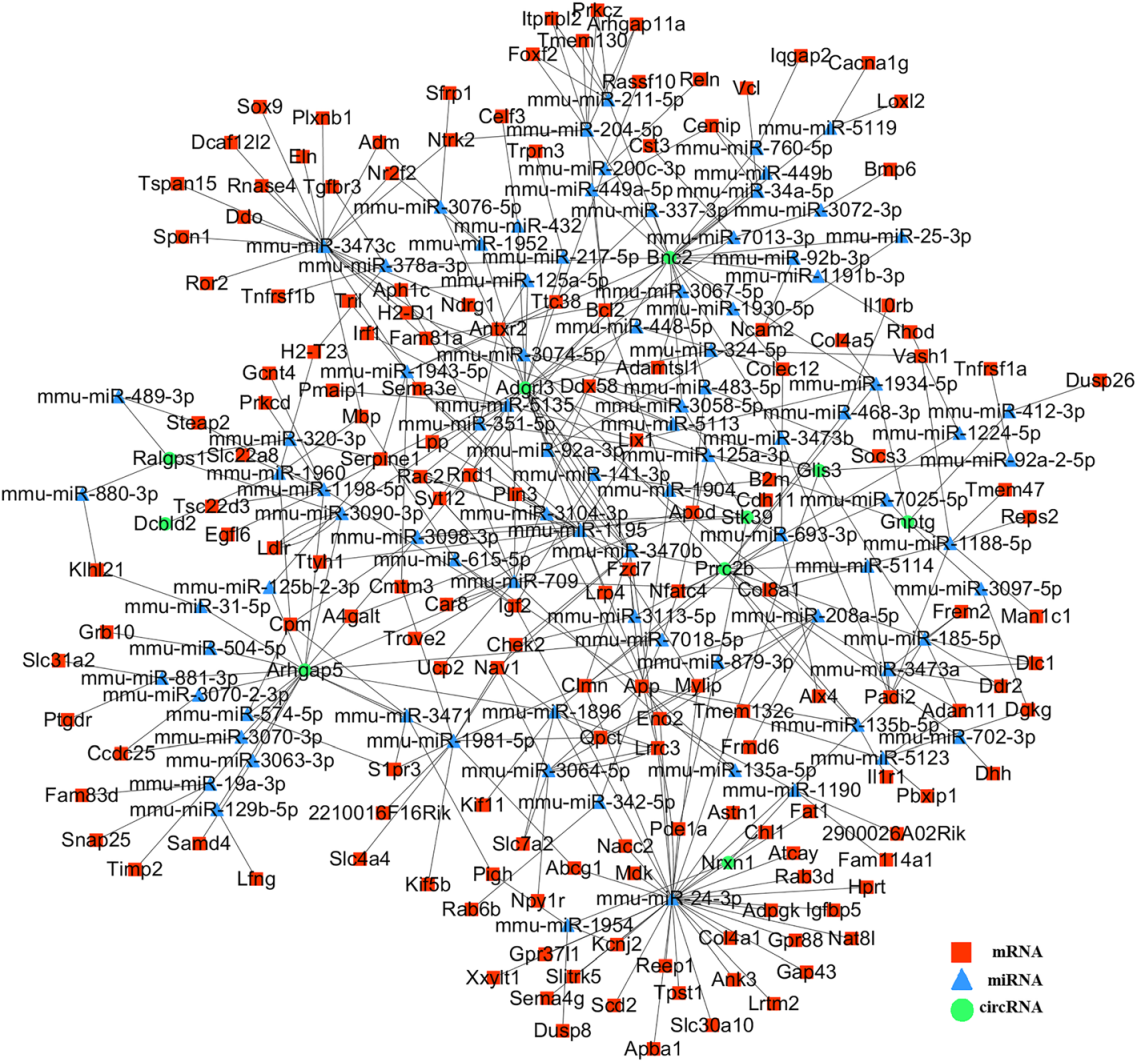
GO enrichment analysis of the mRNA in the co-expression network. The GO analysis (*P*-value < 0.05 and supporting reads number > =2) contains two domains: Biological Process and Cellular Component

To further detect the regulation of these circRNAs in NSC differentiation, we constructed a co-expression network for thedifferentially expressed circRNA and mRNA molecules. We first downloaded the database on mirTarBase and found the miRNA target sites verified by experiment for the differentially expressedmRNA. For the lack of the validatedfunction of circRNAs, we could not find their biological function such as their targeted miRNA or co-expressed mRNA in the existing database. To find their potential connection, miRanda was applied to detected the differentially expressedcircRNAs for their possible miRNA target sites for further study. According to the knowledge, a circRNAhas a junctionsite between the head and tail of its sequence. We need to consider the possibility of binding site on the junction site. Foridentifying possible miRNA target sites for circRNAs, we traced back to the sequences of their cognate genes. Then, we copied 100-bp reads from the start site and added them to the end of the sequence to assemble a pseudo circRNA sequence because of the cyclic further of circRNAs.Next, we appliedmiRanda for predicting circRNAbinding sites with miRNA. The results were filtered by setting stricter threshold with miRanda score > 150 and minimum free energy < − 20. By this analysis pipeline, we obtained 492miRNA target sites for these circRNAs. Based on the above results, we constructed a circRNA-mRNA co-expression network (Fig. 6). We listed the mRNA which shared the same miRNA target sites with circRNA. Then, we built the co-expression network by using Cytoscape.

To further find the possible regulatory roles of the RNA (both circRNA and mRNA), we first applied GO enrichment analysis on all the mRNA genes in the reconstructed co-expression network (Additional file 1: Figure S6). We found that these mRNA genes were enriched in cell adhesion, multicellular organism development, long-term synaptic potentiation, regulation of cell proliferation, regulation of osteoblast proliferation, long-term memory, neuron migration, neuromuscular junction development, cell differentiation and dozens of GO Biological Process terms closely associated with NSC differentiation.And these mRNA genes were also enriched in axon, cell surface, neuronal cell body, dendrite, cell junction, neuromuscular junction, neuron projection and cell-cell junction as GO Cellular Component terms. These significant enriched GO terms are associated with NSC differentiation, suggesting mRNAs in the co-expression network involved in the regulation during NSC differentiation. As for the KEGG Pathway analysis, Axon guidance(mmu04360) was most significantly enriched. These results verified the function of mRNA in the co-expression network, which further indicates the regulatory function of circRNAs in neural activity. However, to better study on these circRNAs, we need to classify the mRNA in the network for their different expressed patterns during the differentiation.

## Discussion

**Fig. 7:**
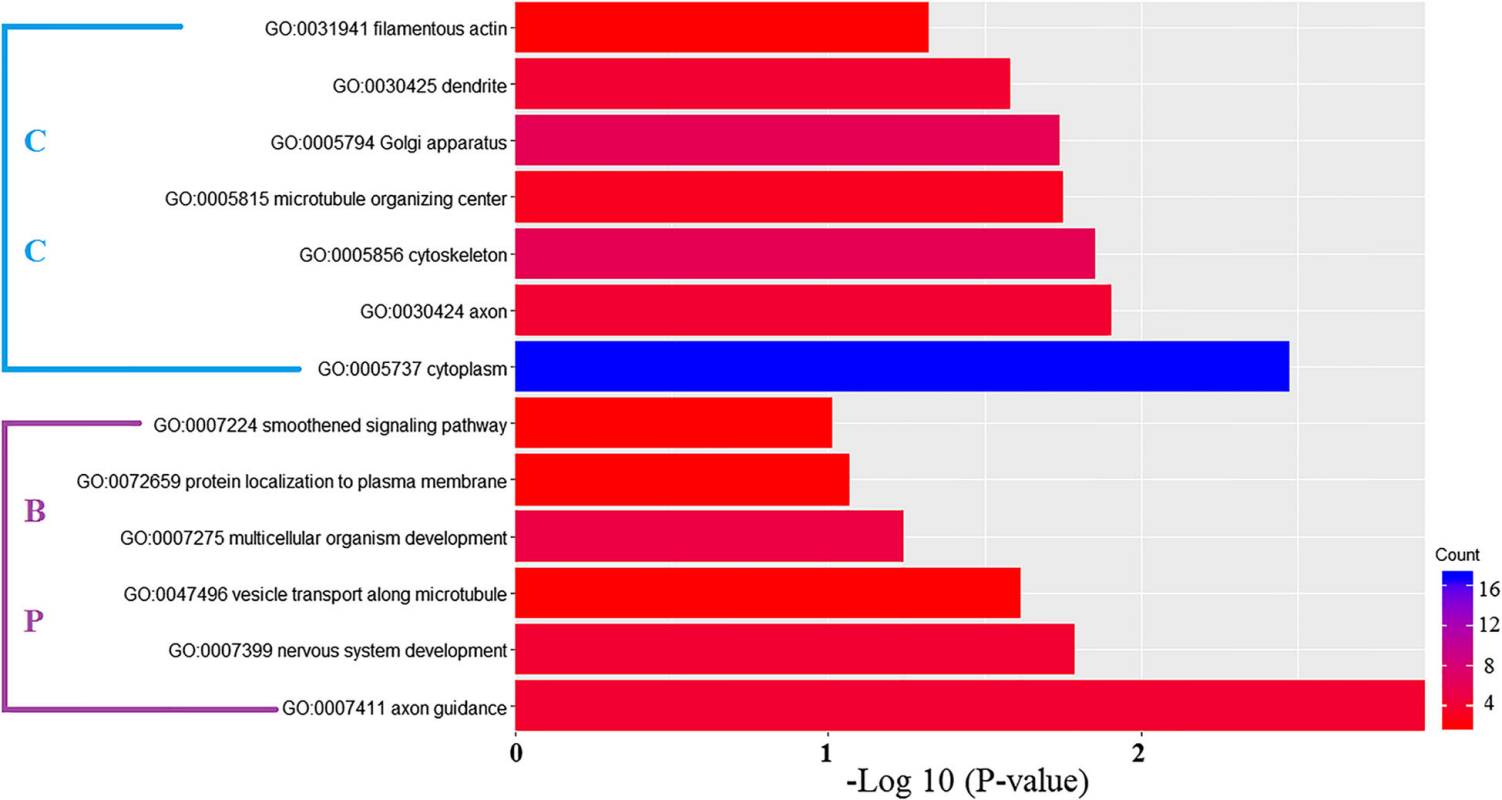
GO enrichment analysis of the mRNA in the co-expression network in the Pattern 4. The GO analysis (*P*-value < 0.05 and supporting reads number > =2) contains two domains: Biological Process and Cellular Component

Similar to circRNAs, weclassifiedthe mRNAs in the co-expression network into four different patterns. Pattern 1 represents mRNA expression increase during the differentiation (expression profile in 0d.nsc < 2d.nscand 2d.nsc < 6d.nsc). Pattern 2 represents an opposite trend of Pattern 1 (expression profilein 0d.nsc > 2d.nscand 2d.nsc > 6d.nsc). Pattern 3 represents mRNAs with the highest expression in the group 2d.nsc (expression profile in 0d.nsc < 2d.nsc and 2d.nsc > 6d.nsc), and Pattern 4 is an opposite trend from Pattern 3 (expression profilein 0d.nsc > 2d.nscand 2d.nsc < 6d.nsc). In our GO enrichment analysis of the mRNAsfrom the fourdifferent patterns, we found that Pattern 4 had the most interesting result (Fig. 7 and Additional file 1: Figure S7). In Pattern 4, mRNAs were enriched in nervous system development,multicellular organism development andcell differentiation (GO:Biological Process), axon, cell junction, synapse and cell projection (GO:Cellular Component). These GO termsare much related to neural activity. It is worth noting that theseenriched GO termsin Pattern 4ranked the topin the analysis rather than some irrelevant terms appeared in the top list for other patterns. The result implies that the mRNAs with expression profile in0d.nsc > 2d.nscand 2d.nsc < 6d.nscmight regulate the differentiation, which coincided with the phenomenon that group 2d.nsc had the least mRNAs at 0.1, 10 and 100 thresholds (Additional file 1: Table S1). In the GO enrichment analysis and KEGG pathway analysis, mRNAs expressed in group 2d.nsc had better results than the other two groups;again, this isconsistentwith the above phenomenon.These results further validated that mRNAs in the Pattern 4 might have regulatory function in differentiation and their co-expression network with circRNAsmight be critical in the process as well. In the NSCs, circRNAs had an overall high expression in the medium differentiation stage while mRNA had the opposite trend.Their specific function duringdifferentiation will need be experimentally investigated in future.

Interestingly,for the Pattern 1 (expression in 0d.nsc < 2d.nsc < 6d.nsc), the top enriched GO termsin Biological Process included negative regulation of ossification, negative regulation of cell proliferation, negative regulation of mitotic cell cycle, negative regulation of osteoblast proliferation,central nervous system developmentpositive regulation of axonogenesis and cell differentiation. These enriched GO terms suggestedmRNA genes in Pattern 1 had strong connection with differentiation and CNS development. They have regulatoryroles in suppressing proliferation and promoting differentiation of NSCs. However, this resultdid not stand out from the differentially expressed mRNAs of the previous Pattern 1 (Additional file 1: Figure S4). These GO terms did appear in the top listbut the bottom from enriched Biological Process terms.In Pattern 4, we observed that most enriched GO terms in Biological Process had a better performance with stronger connection with differentiation. Taken together,circRNAs and mRNA in our co-expression network had closer relationships with NSCs differentiation and showed potential regulatory functions. In future work, we will generate more experimental datasetto gain higher confidence in the expression patterns of thesecircRNAs and the linear mRNA co-expression network.

Due to the limitation of technology and funding problems, we did not apply further biological experiments to validate the results. The judgement of some GO enriched analyses was set as *P*-value rather than adjusted P-value because the number of RNAs is small. Besides, we also need perform more replicates for high statistical confidence in the further study.

## Conclusions

In this study, we first found that 12 circRNA expression was modulated during mouse NSC differentiation. Then, weconstructed one specific co-expression network for the differentially expressed circRNAs and mRNAs. The GO enrichment analysisrevealed that circRNAs mightplay regulatory roles during NSCs differentiation. In addition, we found that the expression profiles of circRNAswere different from mRNAs. There were more circRNA in 2d.nsc than in 0d.nsc and 6d.nsc while there were fewer mRNAs in 2d.nsc than in 0d.nsc and 6d.nsc. In addition, circRNA in Pattern 3 had closer connections with neural activity while mRNA in the opposite Pattern 4 had closer relationships. Based on this observation, we hypothesized that there are complexcircRNA-mRNA regulation mechanisms that act on NSC differentiation.This study is the first to investigate the circRNA expression profiles during the NSCs differentiation in mice and we will keep studying on this.

## Methods

### NSC differentiation and RNA extraction

To identify the biological function of circRNAsduring the differentiation of NSCs, we first separated and cultured NSCs from the wild-type C57BL/6 J mouse fetal cerebral cortex. NSCs from mouse fetal cerebral cortex at E15.5 days were then dissociated by trituration. After centrifugation at 1000 r/min for 4 min, cells were cultured and seeded in culture flask treated with regent Poly-L-ornithine hydrochloride (GIBCO) and Fibronectin (GIBCO). This procedure could help NSCs adherent to the surface of the flask. Cells were maintained at 37 °C in the incubator for 2 weeks, during which most alive cells in the flask were NSCs with the help of the specific medium selection. The recipe of proliferation medium is DMEM/F-12(1:1) (GIBCO) with 2% B27 (GIBCO), 1% Penicillin-Streptomycin (GIBCO) and 25 ng/ml bFGF (Mouse basic fibroblast growth factor, GIBCO). Next, approximately 2 × 10^6^ cells/mL were seeded in six separate flasks. We divided these flasks into 3 groups. Two flasks of NSCs were kept their undifferentiated state during a six-dayculturing. They did not differentiate and we named this group as group 0d.nsc. Another replicate of flasks was induced to differentiate on day 4. We induced them to differentiate into neuron and neurogliocyte cells by wiping off the differentiation-suppress regent bFGF. These NSCs differentiated in 2 days and we marked these as group 2d.nsc. The rest of the flasks differentiated at the first time of the experiment and kept differentiating in 6 days. We named this group as 6d.nsc. We extracted the total RNA of these six flasks on day 6. Finally, we extracted total RNA from the differentiate cells at three different time points. Total RNA was extracted by using Total RNA Kit (TIANGEN, co. LTD., China), which used RNase-free spin column to isolate RNA from the cells.

These RNAs were stored in liquid nitrogen and sent to Beijing Genomics Institute (BGI) for transcriptomic assay (RNA-Seq, paired end, 100-bp). A total of six groups of data were obtained andthere were approximately 43 million reads for each sample.

### Library preparation and sequencing

End-repair and adaptor ligation were processed by using Illumina’s TrueSeq Total RNA Library Prep Kit. Size of 250–300 bp cDNA fragments were separated and then PCR-amplified for about 20 cycles to build the library. After further purification and detection, the RNA-Seq was generated by using Illumina Hi-Seq 4000 sequencer.

### Identify differential expression genes

RNA-Seqshort reads were aligned to the mouse reference genome (GRCm38 mm10) using TopHat (version 2.1.1). On average, approximately 85.7% (minimum 82.3%; maximum 88.9%) of RNA sequencing reads could successfully mapped to the mouse reference genome. By using Cufflinks, we assembledthe transcripts, which had been annotated in database GENCODE. Then we employed the tool Cuffdiff (version 2.2.1) to identify the differential expression genes and transcripts between the different time courses. Stricter standard Q-values were used and the cutoffs were set as below 0.05.

### CircRNA detection pipeline

The RNA-Seq data were initially filtered through TopHat by mapping to the reference genome. The canonical splicing sites were detected and most reads were mapped. Then, the unmapped reads, were processed by UROBORUS (version 1.0.0, http://uroborus.openbioinformatics.org/en/latest/).

### Functional pathway analysis

GO terms have been commonly used forenrichment analysis of genes of interest (e.g.,differentially expressed genes in this study). We performed functional annotation clustering by using the Database for Annotation, Visualization and IntegratedDiscovery (DAVID version 6.8) [50]. The background gene set consists of all expressed in mouse muscles andthe *P*-value was set< 0.05. We also performed enrichment analysis using KEGGpathways and the P-value was set < 0.05 [51].

### Prediction of miRNA target sites on circRNAs

By using miRanda, the database for predicted microRNA targets and target downregulation scores, we obtained the miRNA target sites for circRNAs. We assembled the sequences of circRNAs by following two steps: (1) assembled the annotated exons from the differentially expressedcircRNAs, and (2) copied 100-bp reads from the startof the sequence and added them to the end of the sequence. Then, we applied miRanda on these sequences to predict miRNA target sites.We set the complementarity score threshold to 150.0 and set the complementarity energy threshold to − 20.0 kcal/mol to improve the prediction accuracy.

### Visualization of the circRNA-mRNA co-expression network

By combing the verified miRNA targets from mirTarBase and the predicted miRNA targets from miRanda, we constructed the circRNA-mRNA co-expression networkby using Cytoscape (version 3.5.1), an open source software platform for visualizing complex networks. The network contains three types of nodes (circRNA, miRNA, mRNA), and the nodes mRNA and circRNAshared the same miRNA targets.The construction of the co-expression network included three steps: (1)identification of the those up- or down-regulatedcircRNAs, (2)appliedmiRanda to predict miRNA target sites on differentially expressedcircRNAs, and (3)obtained the list ofdifferentially expressedmRNAs and selected their miRNA targetsfrom mirTarBase database with experiment support.

## Additional file

Additional file 1 : **Figure S1**. Heatmap and correlation matrix for top 200 expressed genes in different time course. **Figure S2**. Profiling of differentially expressed mRNAs during NSC differentiation. **Figure S3**. GO enrichment analysis and KEGG PATHWAY analysis on the differentially expressed genes in differentiation. **Figure S4**. GO enrichment analysis and KEGG PATHWAY analysis on differentially expressed mRNA in Pattern 1, 2 and 3. **Figure S5**. Circular/linear expression ratio between these circRNA and their parental genes. **Figure S6**. GO enrichment analysis on mRNA in the co-expression network. **Figure S7**. GO enrichment analysis and KEGG PATHWAY analysis mRNA in the co-expression network in Pattern 1, 2 and 3. **Table S1**. Number of genes expressed at various FPKM thresholds in different group. **Table S2**. Number of Up-regulated genes and Down-regulated genes in differential analysis. **Table S3**. GO analysis of the differentially expressed mRNA in Pattern 4#. **Table S4**. Number of circRNA expressed in differentiation. (PDF 1228 kb)

## Abbreviations

circRNAs: Circular RNAs
CNS: Central nervous system
DAVID: Database for Annotation, Visualization and Integrated Discovery
GO: Gene Ontology
KEGG: Kyoto Encyclopedia of Genes and Genomes
miRNA: microRNA
NSCs: Neural stem cells
RBPs: RNA-binding proteins
RNA-Seq: Paired-end ribominus RNA sequencing
UCSC: University of California Santa Cruz
