# Correction to: circular RNA expression profiles during the differentiation of mouse neural stem cells

**DOI:** 10.1186/s12918-019-0682-2

**Published:** 2019-01-24

**Authors:** Qichang Yang, Jing Wu, Jian Zhao, Tianyi Xu, Zhongming Zhao, Xiaofeng Song, Ping Han

**Affiliations:** 1 Department of Biomedical Engineering, Nanjing University of Aeronautics and Astronautics, Nanjing, 211106 Jiangsu China; 2 Center for Precision Health, School of Biomedical Informatics, The University of Texas Health Science Center at Houston, Houston, TX 77030 USA; 3 Department of Biomedical Informatics, Vanderbilt University Medical Center, Nashville, TN 37203 USA; 4 The First Affiliated Hospital with Nanjing Medical University, Nanjing, 210019 Jiangsu China

**Correction to: BMC Systems Biology 2018, 12(Suppl 8):128**

**https://10.1186/s12918-018-0651-1**

It was highlighted that the original article [1] contained a mistake in the grant number in the Funding section of the Declarations, and in the legend of Fig. 6. This Correction article shows the incorrect and correct version of the Funding and the legend of Fig. 6.

## Incorrect:

**Fig. 6 legend**

GO enrichment analysis of the mRNA in the co-expression network. The GO analysis (*P*-value < 0.05 and supporting reads number > =2) contains two domains: Biological Process and Cellular Component.

**Funding**

Publication of this article was funded by the National Natural Science Foundation of China (No.81270700 and No.81571223). Z.Z was partially supported by National Institutes of Health grants (R21CA196508 and R01LM012806). The funder had no role in the study design, data collection and analysis, decision to publish, or preparation of the manuscript.

## Correct:

**Fig. 6 legend**

circRNA-mRNA co-expression network. Red box represents mRNA; blue triangle represents miRNA, and green circle represents circRNA.

**Funding**

Publication of this article was funded by the National Natural Science Foundation of China (No.81270700 and No. 61571223). Z.Z was partially supported by National Institutes of Health grants (R21CA196508 and R01LM012806). The funder had no role in the study design, data collection and analysis, decision to publish, or preparation of the manuscript.
