# Supplementary material for: Circular RNA expression profiles during the differentiation of mouse neural stem cells
Source: BMC Syst Biol. 2018 Dec 21;12(Suppl 8):128. doi: 10.1186/s12918-018-0651-1 (PMC6302452; doi:10.1186/s12918-018-0651-1)
Supplement: Supplementary file 1 — : Figure S1. Heatmap and correlation matrix for top 200 expressed genes in different time course. Figure S2. Profiling of differentially expressed mRNAs during NSC differentiation. Figure S3. GO enrichment analysis and KEGG PATHWAY analysis on the differentially expressed genes in differentiation. Figure S4. GO enrichment analysis and KEGG PATHWAY analysis on differentially expressed mRNA in Pattern 1, 2 and 3. Figure S5. Circular/linear expression ratio between these circRNA and their parental genes. Figure S6. GO enrichment analysis on mRNA in the co-expression network. Figure S7. GO enrichment analysis and KEGG PATHWAY analysis mRNA in the co-expression network in Pattern 1, 2 and 3. Table S1. Number of genes expressed at various FPKM thresholds in different group. Table S2. Number of Up-regulated genes and Down-regulated genes in differential analysis. Table S3. GO analysis of the differentially expressed mRNA in Pattern 4#. Table S4. Number of circRNA expressed in differentiation. (PDF 1228 kb) [file 12918_2018_651_MOESM1_ESM.pdf]

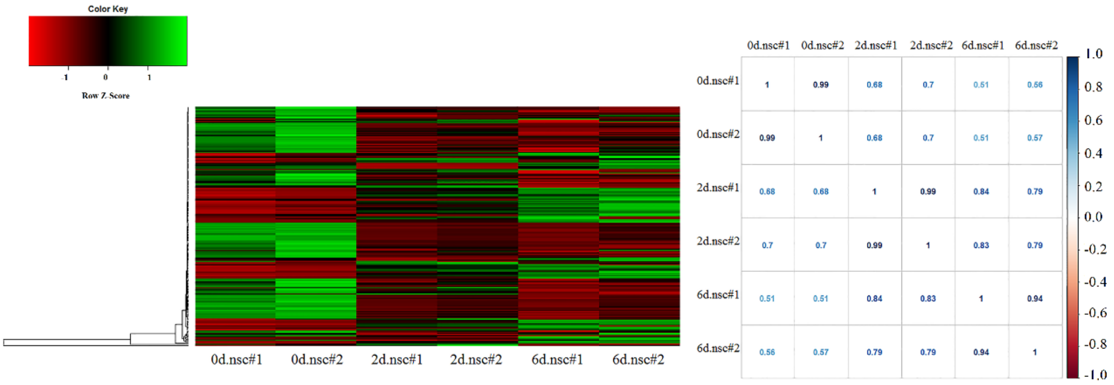

Figure S1: Heatmap and correlation matrix for top 200 expressed genes in different time course. (A) Heatmap of expression profiles for top 200 expressed genes in different groups, red through green color indicates the expression level from low to high. (B) The matrix contains the correlation between different groups, Blue color means high correlation and Red color means weak correlation.

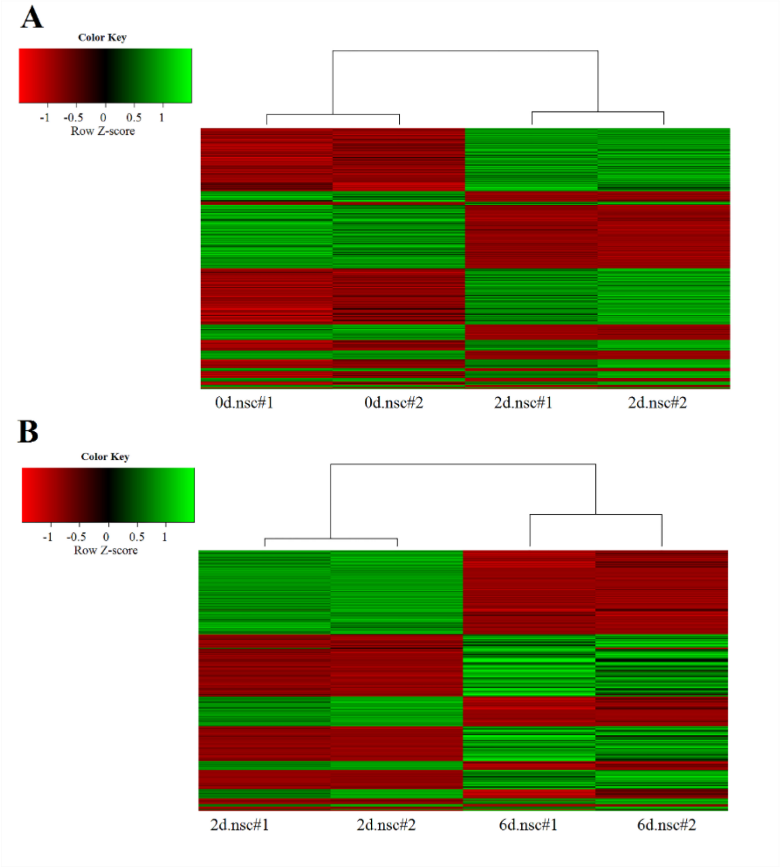

Figure S2: Profiling of differentially expressed mRNAs during NSC differentiation. Heatmap of expression profiles for mRNA that were differentially expressed during NSC differentiation The color bar (red through green) indicates the expression level from low to high. (A) Differentially expressed mRNA in differential expression analysis between groups 2d.nsc and 0d.nsc. (B) Differentially expressed mRNA in differential expression analysis between groups 6d.nsc and 2d.nsc.

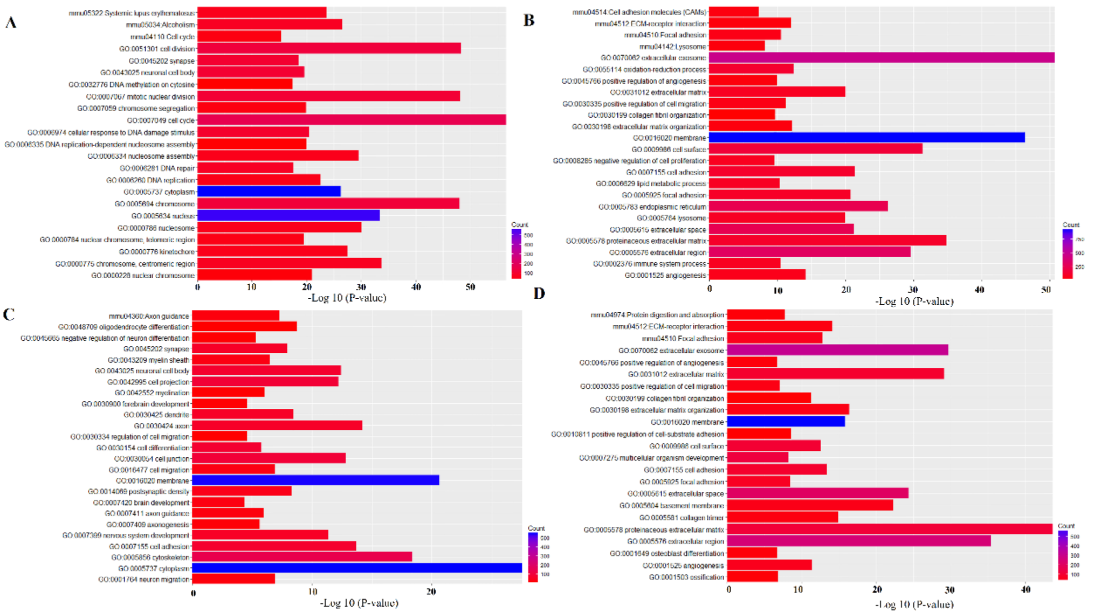

Figure S3: GO enrichment analysis and KEGG PATHWAY analysis on the differentially expressed genes in differentiation. The top 10 GO enriched items in Biological Process and Cellular Component and top 3 enriched KEGG PATHWAY item. (A) Down-regulated mRNA in 2d.nsc versus 0d.nsc. (B) Up-regulated mRNA in 2d.nsc versus 0d.nsc. (C) Up-regulated mRNA in 6d.nsc versus 2d.nsc. (D) Down-regulated mRNA in 6d.nsc versus 2d.nsc.

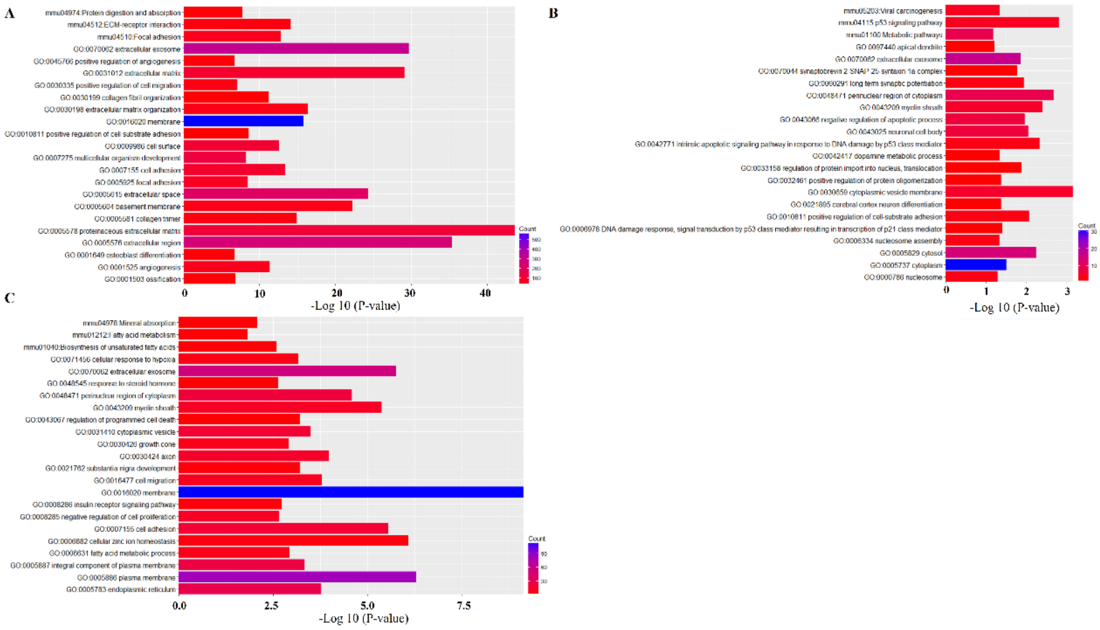

Figure S4: GO enrichment analysis and KEGG PATHWAY analysis on differentially expressed mRNA in Pattern 1, 2 and 3. The top 10 GO enriched items in Biological Process and Cellular Component and top 3 enriched KEGG PATHWAY item. (A) Analysis on Pattern 3. (B) Analysis on Pattern 2. (C) Analysis on Pattern 1.

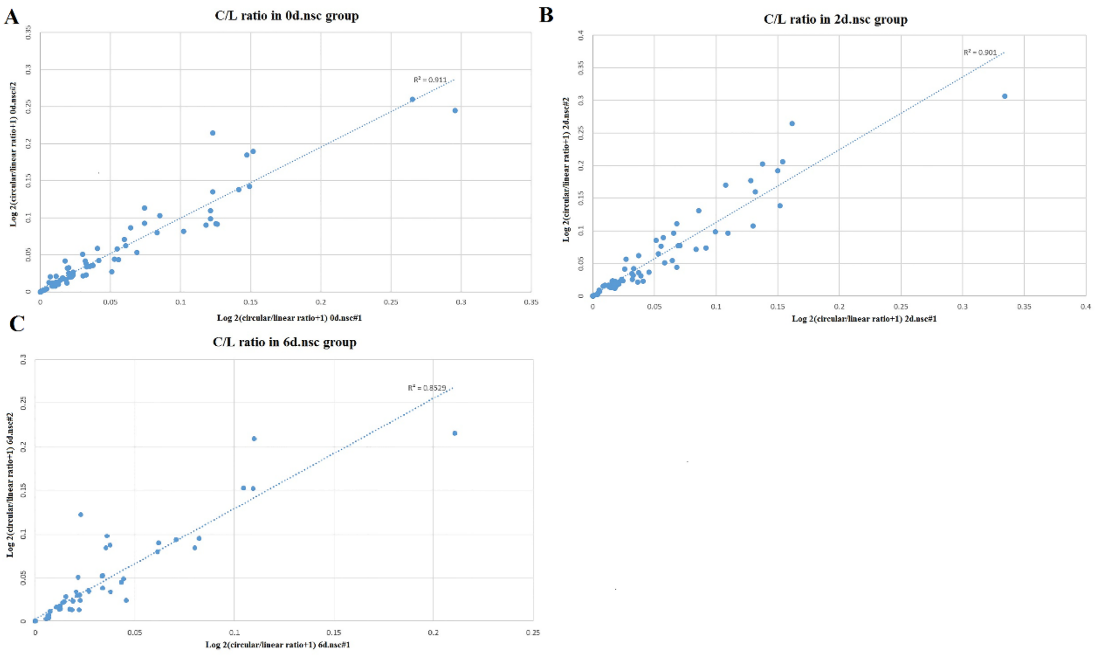

Figure S5: Circular/linear expression ratio between these circRNA and their parental genes. Circular/linear expression ratio between these circRNA and their parental genes in different replicates. (A) C/L ratio in 0d.nsc replicates. (B) C/L ratio in 2d.nsc replicates. (C) C/L ratio in 6d.nsc replicates.

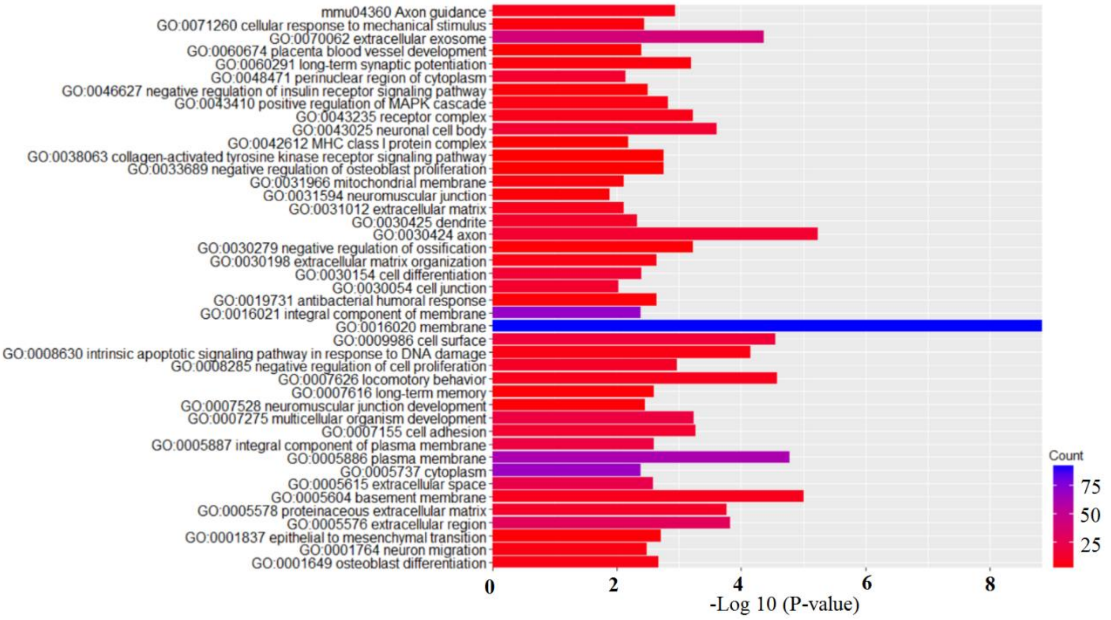

Figure S6: GO enrichment analysis on mRNA in the co-expression network. GO enrichment analysis on mRNA in the co-expression network.

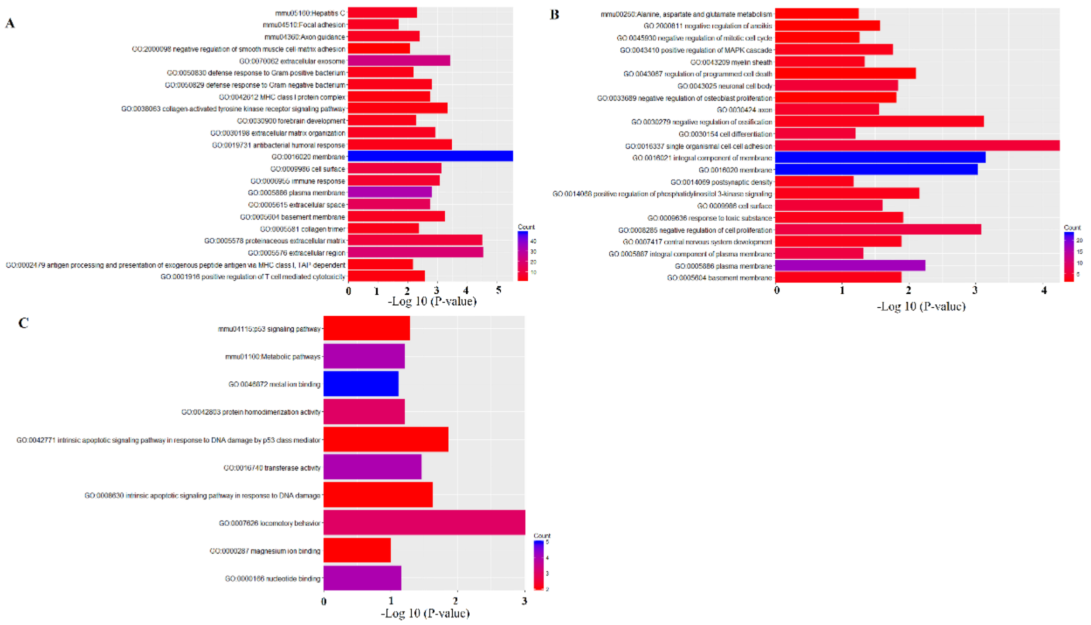

Figure S7: GO enrichment analysis and KEGG PATHWAY analysis mRNA in the co-expression network in Pattern 1, 2 and 3. The top 10 GO enriched items in Biological Process and Cellular Component and top 3 enriched KEGG PATHWAY item. (A) Analysis on Pattern 3. (B) Analysis on Pattern 2. (C) Analysis on Pattern 1.

Table S1: Number of genes expressed at various FPKM thresholds in different group.

	0d.nsc#1	0d.nsc#2	2d.nsc#1	2d.nsc#2	6d.nsc#1	6d.nsc#2
FPKM>0.1	13215	13214	13175	13175	13063	13063
FPKM>1	11435	11447	11405	11359	11271	11252
FPKM>10	5290	5389	5010	5028	5376	5485
FPKM>100	462	498	407	437	442	486

Table S2: Number of Up-regulated genes and Down-regulated genes in differential analysis

	2d.nsc versus 0d.nsc	6d.nsc versus 2d.nsc
Up-regulated	1703	1136
Down-regulated	1214	1224

Table S3: GO analysis of the differentially expressed mRNA in Pattern 4#

Biological Process Term	Count	P-value
GO:0007067 mitotic nuclear division	15	1.54E-08
GO:0007049 cell cycle	19	5.25E-07
GO:0007409 axonogenesis	9	1.16E-06
GO:0051301 cell division	14	3.60E-06
GO:0007411 axon guidance	9	1.37E-05
GO:0007399 nervous system development	13	2.09E-05
GO:0007018 microtubule-based movement	6	2.65E-04

GO:0048709 oligodendrocyte differentiation	4	0.001928
GO:0001764 neuron migration	6	0.002162
GO:0045665 negative regulation of neuron differentiation	5	0.002319
GO:0045773 positive regulation of axon extension	4	0.003084
GO:0007017 microtubule-based process	4	0.00331
GO:0051310 metaphase plate congression	3	0.003327
GO:0007059 chromosome segregation	5	0.004105
GO:0007051 spindle organization	3	0.005935
GO:0007064 mitotic sister chromatid cohesion	3	0.006695
GO:0006915 apoptotic process	11	0.009023
GO:0007088 regulation of mitotic nuclear division	3	0.00922
GO:0000070 mitotic sister chromatid segregation	3	0.012104
GO:0090316 positive regulation of intracellular protein transport	3	0.013142
GO:0001578 microtubule bundle formation	3	0.016477
GO:2000463 positive regulation of excitatory postsynaptic potential	3	0.01766
GO:0007275 multicellular organism development	15	0.01814
GO:0019233 sensory perception of pain	4	0.018791
GO:0007165 signal transduction	17	0.0206
GO:0030951 establishment or maintenance of microtubule cytoskeleton polarity	2	0.021742
GO:0032387 negative regulation of intracellular transport	2	0.021742
GO:0048663 neuron fate commitment	3	0.022739
GO:0000086 G2/M transition of mitotic cell cycle	3	0.024092
GO:0014051 gamma-aminobutyric acid secretion	2	0.028884
GO:0000226 microtubule cytoskeleton organization	4	0.03324
GO:0030010 establishment of cell polarity	3	0.034436
GO:0045143 homologous chromosome segregation	2	0.035975
GO:0000122 negative regulation of transcription from RNA polymerase II promoter	11	0.041004
GO:0006887 exocytosis	4	0.041622
GO:0047497 mitochondrion transport along microtubule	2	0.043015
GO:0030819 positive regulation of cAMP biosynthetic process	3	0.049773
Cellular Component Term	Count	P-value
GO:0005737 cytoplasm	81	1.05E-08
GO:0005856 cytoskeleton	27	7.35E-08
GO:0005874 microtubule	14	6.34E-07
GO:0005694 chromosome	12	3.64E-05
GO:0030425 dendrite	14	4.93E-05
GO:0030424 axon	12	7.02E-05
GO:0000922 spindle pole	7	1.11E-04
GO:0005634 nucleus	65	1.16E-04
GO:0043025 neuronal cell body	13	4.45E-04
GO:0005871 kinesin complex	5	5.11E-04
GO:0000775 chromosome, centromeric region	7	5.22E-04
GO:0015630 microtubule cytoskeleton	7	9.47E-04
GO:0045202 synapse	12	9.94E-04

GO:0000776 kinetochore	6	0.001686
GO:0030054 cell junction	14	0.001878
GO:1990023 mitotic spindle midzone	3	0.002654
GO:0097431 mitotic spindle pole	3	0.00373
GO:0048471 perinuclear region of cytoplasm	13	0.00396
GO:0042995 cell projection	13	0.004956
GO:0005815 microtubule organizing center	6	0.005076
GO:0005635 nuclear envelope	6	0.005491
GO:0014069 postsynaptic density	7	0.007362
GO:0019013 viral nucleocapsid	3	0.010592
GO:0043209 myelin sheath	6	0.01218
GO:0000785 chromatin	5	0.012627
GO:0016020 membrane	64	0.013296
GO:0043234 protein complex	11	0.014491
GO:0005794 Golgi apparatus	16	0.022443
GO:0005795 Golgi stack	3	0.024336
GO:0030426 growth cone	5	0.02706
GO:0000942 condensed nuclear chromosome outer kinetochore	2	0.028181
GO:0031262 Ndc80 complex	2	0.028181
GO:0045298 tubulin complex	2	0.028181
GO:0043005 neuron projection	8	0.030901
GO:0016363 nuclear matrix	4	0.032072
GO:0005813 centrosome	8	0.036308
GO:0034991 nuclear meiotic cohesin complex	2	0.041974
GO:0008278 cohesin complex	2	0.041974
GO:0072686 mitotic spindle	3	0.044177

Table S4: Number of circRNA expressed in differentiation.

	0d.nsc#1	0d.nsc#2	2d.nsc#1	2d.nsc#2	6d.nsc#1	6d.nsc#2
Number	4649	4918	4593	4709	3158	3545
